# Pancreaticoduodenectomy with combined superior mesenteric artery and vein resection for isolated lymph node recurrence of colon cancer: a case report

**DOI:** 10.1097/RC9.0000000000000562

**Published:** 2026-06-04

**Authors:** Takashi Miyata, Shota Motoyama, Hisashi Nishiki, Hideto Fujita, Daisuke Sakamoto, Hiroyuki Takamura

**Affiliations:** a Department of General and Digestive Surgery, Kanazawa Medical University Hospital, Ishikawa, Japan; b Department of Cardiovascular Surgery, Kanazawa Medical University Hospital, Ishikawa, Japan

**Keywords:** colorectal cancer, pancreaticoduodenectomy, superior mesenteric artery, superior mesenteric vein

## Abstract

**Introduction:**

Pancreatoduodenectomy (PD) with combined superior mesenteric artery (SMA) resection is considered extremely invasive and is rarely indicated, even for pancreatic or biliary tract malignancies, as it seldom results in favorable long-term outcomes. To date, no reports have described PD with combined SMA resection performed for isolated lymph node recurrence of colorectal cancer (CRC).

**Case presentation:**

A 68‑year‑old man underwent laparoscopic right hemicolectomy followed by adjuvant chemotherapy for ascending colon cancer. Two years later, a solitary recurrent lymph node was detected on the ventral aspect of the gastrocolic trunk and SMA. After six cycles of FOLFOXIRI plus bevacizumab, the lesion decreased in size with no evidence of additional metastases. The patient subsequently underwent PD with combined resection of the SMA and superior mesenteric vein, with arterial reconstruction using a great saphenous vein graft. Histopathological examination confirmed an R0 resection.

**Discussion:**

Although SMA resection during PD is rarely performed because of its technical complexity and historically poor outcomes, this case illustrates that aggressive surgical management may be justified in highly selected patients with isolated CRC recurrence. The solitary nature of the lesion, favorable response to chemotherapy, and absence of other metastases supported the decision to pursue radical resection with vascular reconstruction.

**Conclusion:**

This case suggests that PD with combined SMA resection may be a feasible treatment option for selected patients with isolated lymph node recurrence of CRC. Further accumulation of cases is needed to clarify optimal management strategies and long-term outcomes for this rare clinical scenario.

## Introduction

Colorectal cancer (CRC) is the third most common malignancy worldwide and the second leading cause of cancer-related mortality^[^1^]^. Despite advances in radical surgery and adjuvant chemotherapy, approximately 30% of patients develop metachronous metastases during follow-up^[^2^]^. Nevertheless, potentially curative resection of isolated metastatic lesions may provide favorable long-term outcomes, even when major procedures such as pancreatoduodenectomy (PD) are required in carefully selected patients^[^3^]^.

HIGHLIGHTS

- Pancreatoduodenectomy with combined superior mesenteric artery (SMA) and superior mesenteric vein resection is extremely rare.
- Isolated lymph node recurrence of colorectal cancer adjacent to the SMA is exceptional.
- Preoperative FOLFOXIRI plus bevacizumab resulted in tumor shrinkage without new lesions.
- Arterial and venous reconstruction using a great saphenous vein graft was successfully performed.
- R0 resection was achieved, and the patient has remained recurrence-free for 18 months.

The initial laparoscopic right hemicolectomy included D3 lymphadenectomy, according to the Japanese Society for Cancer of the Colon and Rectum (JSCCR) guidelines.

While the feasibility of superior mesenteric vein (SMV) resection during PD has been well established, the role of superior mesenteric artery (SMA) resection and reconstruction remains controversial. SMA involvement has traditionally been considered a limit to resection because of the presumed high morbidity and limited survival benefit^[^4–6^]^.

Herein, we report a rare case of isolated metachronous lymph node recurrence from CRC, located adjacent to the SMA. The patient underwent PD with combined SMA resection and arterial reconstruction using a great saphenous vein graft, resulting in complete (R0) resection.

## Case presentation

A 68-year-old man underwent a laparoscopic right hemicolectomy with regional lymph node dissection for ascending colon cancer. Pathological examination revealed a moderately differentiated adenocarcinoma with serosal invasion (T4a), no regional lymph node metastasis (N0), and no distant metastasis (M0), corresponding to stage IIB according to the eighth edition of the UICC TNM classification. The initial surgery involved D3 lymphadenectomy. A total of 24 lymph nodes were retrieved, including #201 (0/11), #202 (0/6), #203 (0/1), #221 (0/5), and #222 (0/1), all of which were negative for metastasis. He received four cycles of CAPOX as adjuvant chemotherapy.

**Figure 1.**
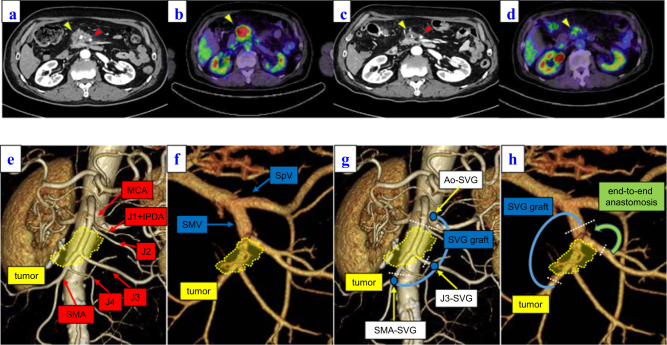
(a) Pre-chemotherapy CT scan showing a solitary recurrent lymph node (yellow arrow) located along the gastrocolic trunk (GCT) and superior mesenteric artery (SMA, red arrow). (b) Pre-chemotherapy PET-CT demonstrating increased fluorodeoxyglucose uptake in the lymph node, with a maximum standardized uptake value (SUVmax) of 13.4 (yellow arrow). (c) After chemotherapy, the lymph node (yellow arrow) decreased in size but continued to abut the SMA (red arrow), with no new lesions detected. (d) After chemotherapy, the SUVmax decreased to 2.1 (yellow arrow), and no additional abnormal uptake was observed. (Ee) Preoperative three-dimensional CT angiogram and schematic illustration of the arterial anatomy. The yellow-shaded area indicates the tumor, which was diagnosed as infiltrating the SMA from the first to third jejunal branches. (f) Preoperative three-dimensional CT angiogram and schematic illustration of the venous anatomy. The yellow-shaded area similarly indicates tumor invasion into the superior mesenteric vein (SMV). (g) Preoperative 3D CT angiogram and schematic of the planned arterial procedure. The yellow shading represents the tumor, the white dotted line indicates the planned SMA resection line, and the blue circle marks the planned anastomosis site between the great saphenous vein (SVG) graft (blue line) and the SMA. (h) Preoperative 3D CT angiogram and schematic of the planned venous procedure. The yellow shading represents the tumor, the white dotted line indicates the planned SMV resection line, the blue line marks the planned SVG graft anastomosis, and the green line indicates the planned direct end-to-end SMV anastomosis.

Twenty-four months after surgery, laboratory tests showed an elevation in CA19-9 from 38.8 to 386.3 U/mL. Follow-up computed tomography (CT) demonstrated a solitary enlarged lymph node located along the gastrocolic trunk and the SMA, adjacent to a surgical clip from the initial operation (Fig. 1a). Positron emission tomography–CT (PET-CT) revealed abnormal uptake of 18 F-fluorodeoxyglucose confined to this lymph node (Fig. 1b). The lesion was clearly separated from the anastomosis and surgical bed, showed a nodal morphology on CT, and demonstrated focal fluorodeoxyglucose uptake consistent with metastatic lymphadenopathy rather than postoperative fibrosis or local extension. Based on these findings, recurrent lymph node metastasis was diagnosed, and six cycles of FOLFOXIRI plus bevacizumab were administered.

After chemotherapy, CA19-9 decreased to 32.8 U/mL, and CT confirmed shrinkage of the recurrent lymph node with no new metastatic lesions (Fig. 1c, Supplemental Digital Content Figure, available at: http://links.lww.com/IJSCR/A62). PET-CT showed marked improvement (Fig. 1d). However, the lymph node was found to be invading the SMA (involving the first to third jejunal branches), the uncinate process of the pancreas, and the SMV (Fig. 1e, f). Therefore, PD with combined SMA and SMV resection was planned. Arterial reconstruction was designed using a great saphenous vein graft to create a bypass between the aorta, J3, and the distal SMA (Fig. 1g). Two sites of SMV resection and reconstruction were also planned (Fig. 1h). The patient provided informed consent for radical surgery.

**Figure 2.**
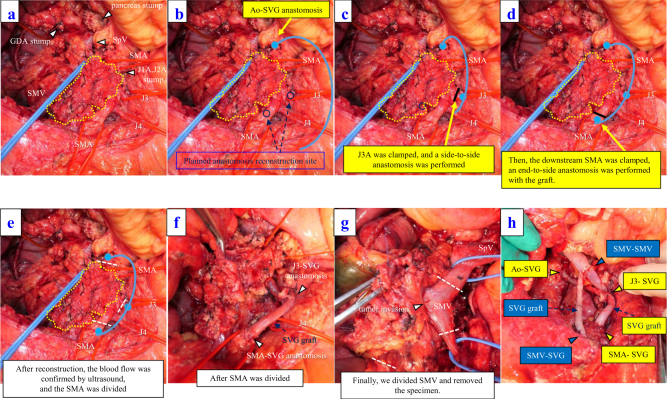
(a) Exposure and encirclement of the SMA, with taping of J3A, J4A, and the distal SMA. (b) Proximal anastomosis between the SVG graft and the aorta. (c) After clamping J3A (black line), a side-to-side anastomosis was performed between the SVG graft and J3A. (d) After clamping the distal SMA (black line), an end-to-side anastomosis was performed between the SVG graft and the SMA. (e) The planned SMA resection line is indicated by the white dotted line. (f) Operative field after SMA division. (g) The planned SMV resection line is indicated by the white dotted line. (h) Operative field after specimen removal and completion of venous reconstruction.

After laparotomy, the SMA was exposed and encircled. The J1, J2, and inferior pancreaticoduodenal artery branches were ligated and divided. Further dissection allowed taping of J3, J4, and the distal SMA (Fig. 2a). As complete resection appeared feasible, PD was performed, leaving the specimen attached only by the SMA and SMV. A cardiovascular surgeon harvested the left great saphenous vein and performed proximal anastomosis to the aorta (Fig. 2b). J3 was clamped, and a side-to-side anastomosis between the graft and J3 was completed before declamping (Fig. 2c). The distal SMA was then clamped, and an end-to-side anastomosis with the graft was performed (Fig. 2d). After confirming adequate blood flow by intraoperative ultrasound, the SMA was divided (Fig. 2e, f). The SMV was then divided, and reconstruction was performed at two sites: one with end-to-end anastomosis and the other using a saphenous vein graft (Fig. 2g, h). Because arterial reconstruction to the jejunal branch (J3) was performed using a great saphenous vein graft, the mobility of the jejunal limb was expected to be limited. To avoid tension on the anastomosis, gastropancreatic anastomosis was selected instead of our usual pancreaticojejunostomy.

The operative time was 908 minutes, and intraoperative blood loss was 1600 mL, requiring transfusion of 720 mL of red blood cells. Postoperatively, the patient developed a pancreatic fistula (Clavien–Dindo IIIa) and diarrhea (Clavien–Dindo II). The pancreatic fistula was managed with percutaneous drainage without the need for reoperation, and the diarrhea was treated conservatively. Antithrombotic therapy consisted of heparin for 5 days, followed by oral aspirin, with no evidence of graft thrombosis. The patient was discharged on postoperative day 34. Postoperative chemotherapy was withheld because he developed postoperative complications and preferred observation rather than additional treatment. Instead, a structured surveillance strategy was adopted, consisting of contrast-enhanced CT every 3–4 months and regular monitoring of tumor markers.

**Figure 3.**
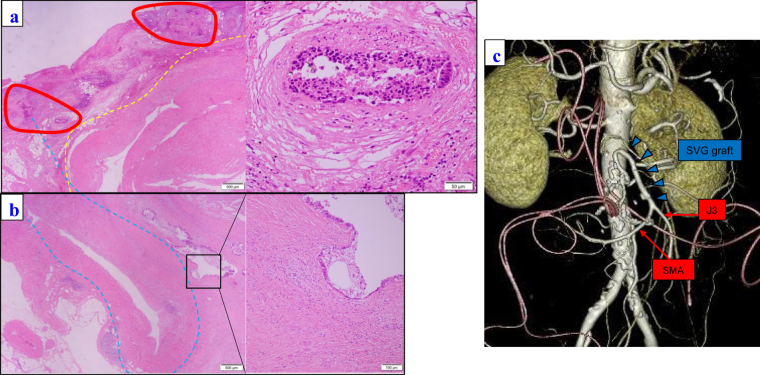
(**a)** Histopathological examination showing multiple perineural infiltrates (red solid lines) within 1 mm of the SMA (yellow dashed line). (b) Tumor infiltration into the adventitial layer of the SMV (light blue dashed line). (c) Postoperative three-dimensional CT angiogram demonstrating the reconstructed SMA arcade using the SVG graft (blue arrows).

Gross examination revealed a whitish, ill-defined mass surrounding the SMA and SMV. Microscopically, the lesion was adenocarcinoma, positive for CDX2 and SATB2, consistent with metastasis from the primary colon cancer. Tumor invasion into the adventitia of the SMA and SMV, as well as into the pancreatic head, was confirmed (Fig. 3a, b). The patient has remained recurrence-free for 18 months postoperatively without additional chemotherapy. Follow-up contrast-enhanced CT at 3 months demonstrated satisfactory blood flow through the reconstructed SMA (Fig. 3c).

## Discussion

The first series of PD with vascular resection was reported by Fortner *et al* in 1977^[^7^]^. Since then, improvements in surgical techniques and perioperative management have made PD with venous resection widely accepted. In contrast, PD with combined SMA resection remains uncommon because of its technical complexity, high morbidity, and the limited survival benefit reported in pancreatic and biliary tract cancers^[^4–6^]^. The present case suggests that, in carefully selected patients with CRC metastases, an aggressive surgical approach may be considered in specific situations.

Arterial resection during PD is technically demanding and has traditionally been regarded as a surrogate marker of biologically aggressive disease, often considered a contraindication to surgery^[^8,9^]^. However, recent studies have demonstrated that PD with arterial resection can be performed safely in selected patients^[^10–12^]^. Two major hypotheses have been proposed regarding the oncological significance of arterial invasion. One posits that arterial invasion reflects aggressive tumor biology, resulting in poor outcomes even after R0 resection because of the high risk of local or distant recurrence^[^13,14^]^. The other suggests that arterial involvement may occur due to anatomical proximity rather than tumor aggressiveness, and that en bloc resection, including the involved artery, may still provide oncological benefit^[^5,14^]^. Importantly, these concepts are derived primarily from pancreatic cancer, and their relevance to metastatic CRC remains uncertain.

For CRC, resection of metastatic lesions – particularly in the liver and lungs – has been shown to improve survival in appropriately selected patients^[^15,16^]^. In contrast, the role of surgery for pancreatic, vascular, or retroperitoneal lymph node metastases is far less established. Chemotherapy alone rarely achieves histological cure in such recurrences, yet several reports have described long-term survival following multidisciplinary treatment, including surgery^[^3,17^]^. In addition, several retrospective series have suggested that R0 resection of para-aortic lymph node metastases may provide a survival benefit in highly selected patients, although the evidence remains limited and heterogeneous^[^18,19^]^. These findings support the notion that, in selected cases, surgical resection may contribute to durable disease control.

The present case demonstrates that PD with combined SMA and SMV resection can be a feasible treatment option for isolated lymph node recurrence of CRC. To our knowledge, no previous English-language reports have described PD with simultaneous SMA and SMV resection and reconstruction for CRC recurrence. In this patient, the recurrence was solitary. Although implantation metastasis was considered the most plausible mechanism, given the location adjacent to the surgical clip, the lesion exhibited a nodal morphology on imaging. Furthermore, PET-CT demonstrated uptake confined to a single lymph node station. Therefore, from a clinical standpoint, the recurrence was managed as an isolated nodal metastasis, and preoperative chemotherapy resulted in tumor shrinkage without new lesions. Alternative strategies, such as systemic chemotherapy alone or palliative approaches, were considered. However, given the solitary nature of the lesion, the favorable response to preoperative chemotherapy, and the patient’s good performance status, surgical resection was deemed appropriate. Given the uncertainty regarding the benefit of lymph node metastasectomy, achieving R0 resection was essential. Therefore, PD with combined vascular resection was performed in collaboration with vascular surgeons, using a great saphenous vein graft for both arterial and venous reconstruction. Pathological examination confirmed negative margins, supporting the validity of this aggressive approach.

Evidence regarding the benefit of adjuvant chemotherapy after PD for lymph node recurrence of CRC remains limited. Although postoperative chemotherapy was considered, it was ultimately withheld based on the patient’s condition. With ongoing advances in systemic therapy – including molecular targeted agents and immune checkpoint inhibitors – further investigation is needed to clarify optimal treatment strategies for lymph node recurrence of CRC.

## Conclusion

PD with combined SMA and SMV resection is rarely indicated because of its technical complexity and historically poor outcomes. However, this case demonstrates that, in highly selected patients with isolated lymph node recurrence of CRC, radical resection, including major vascular reconstruction, can be performed safely and may achieve short-term disease control. While long-term oncologic benefit remains uncertain, this case highlights the feasibility of such an aggressive surgical approach in carefully chosen patients.

## Data Availability

No datasets were generated or analyzed for this case report; therefore, data sharing is not applicable.

## SCARE guidelines

This work has been reported in accordance with the SCARE criteria (Surgical Case Report Guidelines)^[^20^]^.
